# Falls Incidence Compared Between a Multibedded Ward Hospital and a 100% Single-Occupancy Room Hospital: An Uncontrolled Before-After Study

**DOI:** 10.1177/19375867221123607

**Published:** 2022-09-07

**Authors:** Fozia Hussain, Monique van Dijk, Christian Oudshoorn, Erwin Ista

**Affiliations:** 1 Department of Internal Medicine, Division of Nursing Science, Erasmus MC, University Medical Center Rotterdam, Rotterdam, the Netherlands; 2 Department of Internal Medicine, Division of Geriatric Medicine, Erasmus MC, University Medical Center Rotterdam, Rotterdam, the Netherlands

**Keywords:** falls, incident reporting, single rooms, delirium

## Abstract

**Background:**

Single-occupancy patient rooms in hospitals have become popular because of the privacy they offer. A downside, however, is the lack of social control from other patients, which might increase the risk of falls and undetected delirium.

**Aim:**

To study whether the incidence of falls in single-occupancy patient rooms differs from that in multibedded patient rooms. Secondary aims were to establish differences in the context of falls and differences in delirium incidence.

**Methods:**

An uncontrolled observational before-after study was performed during 16 months before and 16 after moving to a hospital with 100% single-occupancy patient rooms. Fall data were retrieved from the hospital incident reporting system. The Delirium Observation Screening Scale (DOSS) was retrieved from the hospital electronic patient data system. Main outcomes were the number of falls per 1,000 patient days, assessed with a Poisson regression analysis, and delirium incidence in fallers.

**Results:**

The incidence of falls was not significantly different between the before period (1.39 falls/1,000 patient days) and the after period (1.38 falls/1,000 patient days; *p* = .924). In the after period, falls in the bathroom were significantly more frequent than in the before period, respectively, 17.2% and 9.4% (*p* = .003). In both periods, one quarter of the patients who fell had been assessed for delirium. In the before period, 57/73 (78%) of those were suspected for delirium (DOSS ≥ 3) versus 37/55 (67%) in the after period (*p* = .225).

**Conclusions:**

In this study, we observed no change in incidence of falls after moving to a hospital with 100% single-occupancy bed rooms.

## Introduction

Patient falls in hospitals continue to be a serious concern, as they may lead to injury, poor vision, dizziness, delirium, prolonged hospital stay, and increased costs (Kisacik & Cigerci, 2019; Najafpour et al., 2019; Röhsig et al., 2019). Furthermore, falls have been significantly associated with increased morbidity and mortality rates (de Souza et al., 2019; Röhsig et al., 2019; Thomas & Balmforth, 2019). The rate of hospital falls varies from 1.3 to 16.9 per 1,000 patient days, depending on patient group, type of hospital, and used reporting systems (de Souza et al., 2019; Röhsig et al., 2019). Delirium is common in hospitalized adults, with reported new onset rates ranging from 11% to 56%, depending on age, cognitive decline rate, and medical status of the patient group concerned (Hshieh et al., 2020). Delirium is a well-known risk factor for in-hospital falls. The authors of a systematic review concluded that the median risk ratio of delirium in patients who fell was 4.5 (range 1.4–12.6; Sillner et al., 2019). These results suggest that falls and delirium are inextricably linked.

Many fall preventions interventions and quality improvement projects have been developed and implemented with aim to reduce in-hospital fall incidences in patients—with varying results (Hempel et al., 2013; LeLaurin & Shorr, 2019; Morris & O’Riordan, 2017). This coincided with the trend for building new hospitals with single-occupancy rooms, in contrast to older hospitals with multibedded patient rooms (Joseph et al., 2018; Ulrich et al., 2008). A systematic review of Taylor et al. describes both advantages (e.g., privacy, infection control) and disadvantages (e.g., feelings of isolation, reduced social interaction) of this trend (Taylor et al., 2018). Multibedded rooms on the one hand are hypothesized to offer social control and shorter nurse response times, which benefit patient falls prevention (Davis et al., 2019). Social control can be seen as the phenomenon that co-patients or their relatives look after another patient and, for instance, call the nurse for assistance when a confused patient attempts to step out of bed. Studies about the effects on falls of the mover from multibedded rooms to single-occupancy rooms on hospital level are limited. Singh et al. (2015) found a significantly increase in fall incidence after the move to single-occupancy rooms during an 18 months period (5.4–15.8; *p* < .001). In two other studies, a temporary increase in falls occurred during 6 months after the move to a hospital with single-occupancy rooms, which normalized after adapting work processes (Maben et al., 2016; Simon et al., 2016). Only Maben et al. studied a large patient population on multiple wards (*n* = 4). The other studies were executed in small hospital (Singh et al., 2015), limited wards (*n* = 3; Simon et al., 2016), or in specific patient group, either patients with dementia or geriatric patients (Blandfort et al., 2020a; Knight & Singh, 2016).

On the other hand, staying in a multibedded room is a risk factor for developing delirium, because of exposure to noise, sleep disruption, stress, lack of privacy, and deterioration in patient–family interaction (Caruso et al., 2014). In a recent study was found that the risk of delirium in frail elderly patients staying in single-occupancy rooms was lower than that in multibedded rooms in geriatric wards (Blandfort et al., 2020b). Further, falls may contribute to the development of delirium, and delirium may lead to a fall. Both falls and delirium share many predisposing risk factors and precipitating events. However, patients with delirium had an increased risk of falls, HR = 4.68 (95% CI [2.58, 8.46], *p* < .001; Blandfort et al., 2020a).

In light of these mixed findings, our primary aim was to estimate the difference in falls incidence between patients in multibed versus single occupancy rooms in an university hospital. A secondary aim was establishing the contextual factors (e.g., time of fall, location) and fall severity and the delirium incidence in patients who fell.

***In light of these mixed findings, our primary aim was to estimate the difference in falls incidence between patients in multibed versus single occupancy rooms in an university hospital***.

## Method

### Design

An uncontrolled before-after study was performed in a large university hospital (1,125 beds). The before data collection period was from January 2017 up to and including April 2018. After the move on May 18, 2018, data were collected in the new hospital with 100% single-occupancy rooms from June 2018 up to and including September 2019.

Study approval was obtained from the Medical Ethics Committee of Erasmus University Medical Center Rotterdam, the Netherlands (MEC-2017-1103).

### Setting and Patients

**Figure 1.**
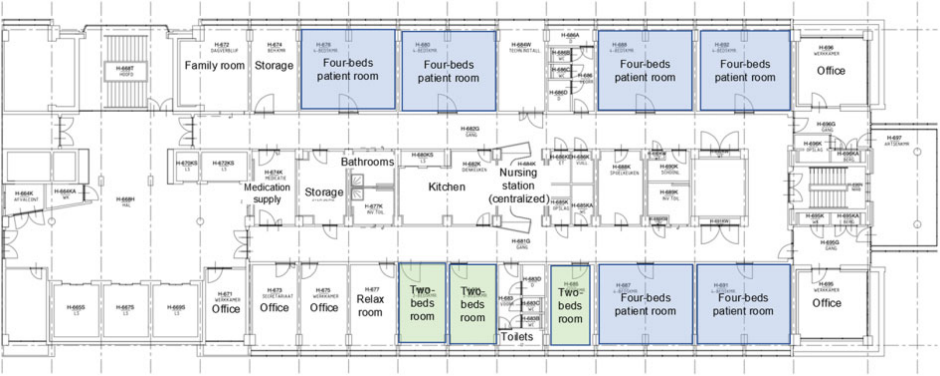
Floor plan of a ward in the former building.

**Figure 2.**
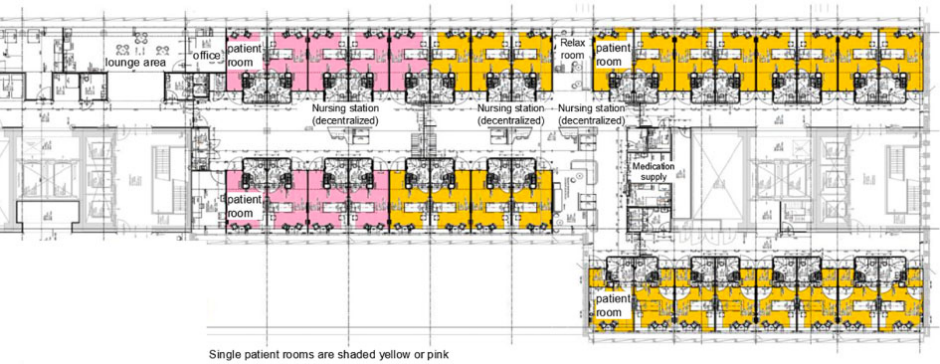
Floor plan of a ward in the new building.

The former building of the university hospital offered two-person and four-person rooms. In some circumstances, a two-person room was used as a single patient room. The new hospital building, replacing the main location and an adult oncology hospital, was designed according to a healing environment concept with exclusively single-occupancy patient rooms. An interdisciplinary team consisting of medical doctors, nurses (nurse)managers, physical therapists, hygienist, microbiologist, architect, and consultancy was involved in the design of wards and patient rooms. The single-occupancy patient rooms were designed with an en-suite bathroom and the possibility for patients to control lighting and windows themselves. A small, soft light shining on the floor on the side of the bed en route to the bathroom provides orientation at night. To facilitate patients’ transfers, all rooms have motorized ceiling lifts. Furthermore, the room has a friendly decor with designer furniture and is painted in a calming color. To facilitate social contact for the patients, there are lounge areas, visiting hours are extended, and a foldout sofa bed provides the opportunity of rooming-in. In the former hospital, patients shared a bathroom and shower located in the corridor, equipped with a pull cord alarm. In the new hospital, each room includes an en-suite bathroom with toilet and shower. Patients wear a wrist alarm to call for assistance if necessary; for hygienic reasons, the bathroom is not equipped with an alarm. Figures 1 and 2 show the floor plan of a ward in the former and one in new hospital (see Online Appendix 1 for photos). In the former hospital, we find centralized nursing units; in the new hospital, we find decentralized nursing stations with computer on wheels available.

The current study is embedded in a larger study examining the effects of the ward layout of the new hospital on patient safety, for example, falls and sleep quality and staff and patients’ perceptions. The included wards of the former hospital were gastroenterology, transplantation unit (kidney/liver), gastroenterological surgery, neurology, oncology-hematology, geriatrics, internal medicine/systemic disease, and pulmonary medicine. In the new hospital, the ward configuration was based on disease-specific care centers. As a result of this policy, we were unable to describe differences between the former and new hospital.

We included all adult inpatients (>18 years) with a length of stay of more than 24 hr. Falls pertaining to patients admitted to the psychiatric wards and the Erasmus MC-Sophia Children’s Hospital, who were not housed in the new building, were excluded. Falls occurring outside patient care areas and falls at the outpatient clinics and daycare clinics were also excluded.

### Outcome Measures

The primary outcome was the incidence of falls per 1,000 patient days, calculated with the equation (reported number of falls/patient day) multiplied by 1,000.

The secondary outcomes were patient-related factors (e.g., age, delirium) and contextual factors (e.g., time of fall, location) of patient falls, and severity of fall-related injury (see Online Appendix 2). The probability of delirium was assessed with the Delirium Observation Screening Scale (DOSS), a 13-point screening tool, completed by a nurse (Schuurmans et al., 2003; see Online Appendix 3). Responses are dichotomous. The highest score of the DOSS is 13; a score of three or higher indicates possible delirium (Scheffer et al., 2011). Patients were diagnosed with delirium if they had at least one DOSS score ≥3, 24 hr before to 48 hr after a fall. Severity of fall-related injury was classified as follows: (1) no harm, (2) minor, (3) moderate, (4) severe, and (5) death. Possible fall-related deaths were checked with data from the hazard research committee.

### Data Collection

The following data on fall incidents were retrieved from the hospital incident reporting system and the electronic health records: patients’ age and sex, length of stay, perceived risk of fall recurrence, time of the day, fall witnessed or not, fall risk screening performed or not, circumstances of the fall, the level of harm (none, low, moderate, severe, and death), any restraint interventions, and DOSS scores. The electronic reporting system or the procedure regarding incident reporting had not changed with the move to the new hospital. Data on patient-days for both periods were retrieved from the electronic health records. To maintain patient confidentiality, the information was pseudoanonymized.

### Statistical Analyses

Data were analyzed using descriptive statistics for demographic information and the characteristics. Frequency and percentages were used for categorical variables. Continuous variables were summarized as mean [standard deviation (*SD*)] or median [range or interquartile range (IQR)] as appropriate. We expressed crude rates of in-hospital falls as rate/1,000 patient days.

Baseline characteristics, age, length of stay, and so on were compared between the before and after periods with a *t* test or Mann–Whitney test. A χ^2^ test was used to compare the percentages of categorical variables between the two periods. A generalized linear model with a Poisson error distribution and a logarithmic link function served to determine the association between study period and falls per 1,000 patient-days. The dependent variable in the Poisson model was the number of falls per 2-month period. The independent variable in the model was study period (before vs. after). The goodness of fit of the Poisson models was evaluated using the deviance statistic (also known as the log-likelihood ratio test). Statistical significance was set at a *p* value of .05. All analyses were performed with IBM SPSS Statistics, Version 25.0, Armonk, NY: IBM Corporation.

## Results

### Patient Characteristics

In total, there were 33,903 admissions in the before period and 32,324 admissions in the after period. The patients’ mean age was, respectively, 58 (*SD* = 17) years and 58 (*SD* = 16) years. Median length of hospital stay in both periods was 3 days (IQR 1–7; *p* = .282; Table 1).

**Table 1.** Patient Characteristics and Falls Information in the Former and New Clinic.

Characteristics	Former Clinic (16 Months)	New Clinic (16 Months)	*p* Value
Number of patients admitted	33,903	32,324	
Males, *n* (%)	18,253 (53.8%)	16,545 (51.2%)	<.001^a^
Age^b^	58 (17)	58 (16)	.001^c^
Length of hospital stay^d^	3 (1–7)	3 (1–7)	.282^c^
In-patients days (*n*)	205,053	198,016	
Total falls (*n*)	286	274	
Falls per 1,000 patients days	1.39	1.38	.924
Total patients with falls (n)	244	232	
Number of single and recurrent falls			.127^e^
- 1 Fall	207 (84.8%)	196 (84.5%)	
- 2 Falls	23 (9.4%)	30 (12.9%)	
- ≥3 Falls	14 (5.7%)	6 (2.6%)	
Chance of recurrence according to incidence *reporter*			0.004^e^
- Impossible	39 (13.6%)	27 (9.9%)	
- Unlikely	19 (6.6%)	8 (2.9%)	
- Even chance	199 (69.6%)	185 (67.5%)	
- Likely	24 (8.4%)	39 (14.2%)	
- Certain	5 (1.7%)	15 (5.5%)	
Hazard documentation fall-related death data	4	1	

^a^ Fisher’s exact test. ^b^ mean (*SD*). ^c^Analysis of variance. ^d^Median (interquartile range). ^e^Pearson χ^2^.

#### Falls

**Figure 3.**
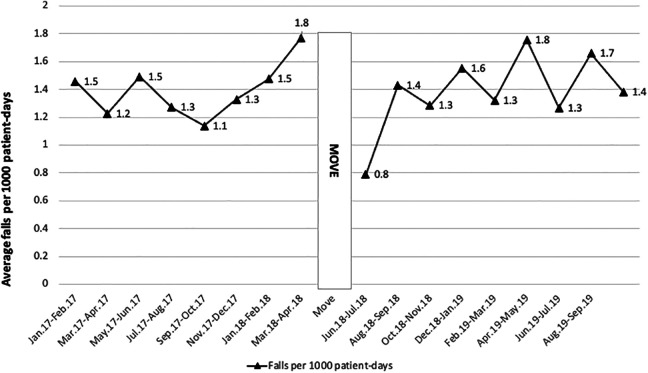
Reported mean numbers of falls per 1,000 patient days per 2 months in former and new clinic.

The line graphs in Figure 3 reflect the mean bimonthly fall rates in the before and after periods. The highest bimonthly fall rate before the move was 1.8 falls/1,000 patient days and the lowest 1.1 falls/1,000 patient days. In the after period, the fall rate ranged from 0.8 to 1.8 falls/1,000 patient days. The lowest fall rate was directly after the move, in the months June/July. Overall, the fall rate did not differ between the two periods (1.39 falls/1,000 patient days before vs. 1.38 falls/1,000 patient days after (−1.5%; 95%CI [−15.9%, 17.1%]; *p* = .924). The goodness of fit of the Poisson models was 16.904 (*df* = 14; *p* = 0.618).

### Characteristics Of Patients Who Fall and Circumstances of Falls

The majority of the patients who fell had been admitted for neurological reasons, respectively, 50% before versus 40% after. Occurrence of falls in relation to the period of the day did not significantly differ between the two periods (*p* = .102). In both periods, most falls were unwitnessed, respectively, 88.8% versus 86.1% (*p* = .338). Circumstances of the falls, however, significantly differed. Patients who fell occurred more often in the en-suite bathrooms than in the shared bathrooms, respectively, 17.2% and 9.4% (*p* = .003). However, fewer patients fell during transfer from bed to chair or wheelchair in the new hospital (40.1%) compared to the former hospital (54.2%; Table 2). In the before period, 10.4% of the falls resulted in moderate or severe injury versus 14.2% in the after period. Two fall-related deaths were reported in the before period versus one in the after period.

The majority of the patients who fell had moderate or severe problems with mobility, respectively, 85.7% before and 75.5% after. In the new hospital, the proportion of patients who fell had no reported mobility problems was 24.5% versus 14.3% in the former hospital.

#### Falls and delirium

More than half of the patients who fell (*n* = 159, 55.6% in the before period vs. *n* = 138, 50.4% in the after period) were moderately or highly restless or confused (Table 2). Delirium was assessed on the day of falling in only 25.5% (73/286) and 20.1% (55/274), for, respectively, the before versus after period. Of those assessed, respectively, 57/73 (78.1%) and 37/55 (73.4%) had a DOSS score of 3 or higher indicating delirium.

**Table 2.** Characteristics of Patients With Falls.

Characteristics	Former Clinic (*n* = 286)	New Clinic (*n* = 274)	*p* Value
Age (years)^a^	65 (15)	64 (15)	.142
Age category, *n* (%)			.017^b^
- 18-49 Years	44 (14.3)	37 (13.5)	
- 50–69 Years	112 (39.2)	139 (50.7)	
- 70+ Years	133 (46.5)	98 (35.8)	
Gender, n (%)			.204^b^
- Male	183 (64.0)	161 (58.8)	
- Female	103 (36.0)	113 (41.2)	
Medical type, *n* (%)			.024^b^
- Surgery	44 (15.4)	41 (15.0)	
- Medicine	32 (11.2)	35 (12.8)	
- Geriatrics	10 (3.5)	7 (2.6)	
- Oncology	37 (12.9)	62 (22.6)	
- Cardiac (including surgery)	15 (5.6)	12 (4.4)	
- Neurology/neurosurgery	144 (50.3)	109 (39.8)	
- Others	3 (1.0)	8 (2.9)	
Time of day, *n* (%)			.102^b^
- Day shift	83 (29.0)	97 (35.4)	
- Evening shift	74 (25.9)	77 (28.1)	
- *Night shift*	129 (45.1)	100 (36.5)	
Fall witnessed (caregiver), yes, *n* (%)	32 (11.2)	38 (13.9)	.338
Location at time of fall, *n* (%)			.003^b^
- Getting up from bed, wheelchair, chair	155 (54.2)	110 (40.1)	
- Bathroom (toileting, shower)	27 (9.4)	47 (17.2)	
- Walking (with and without aid)	98 (34.3)	107 (39.1)	
- Others	6 (2.1)	10 (3.6)	
Injury (reporter), *n* (%)			.344^b^
- No harm	154 (53.8)	135 (49.3)	
- Minor	100 (35.0)	99 (36.1)	
- Moderate	27 (9.4)	30 (10.9)	
- Severe	3 (1.0)	9 (3.3)	
- Death	2 (0.7)	1 (0.4)	
Use of restraint (yes), *n* (%)	43 (15.0)	33 (12.0)	.302
Mobility score, *n* (%)			.010^b^
- Mobile	41 (14.3)	67 (24.5)	
- Moderate mobile	187 (65.4)	159 (58.0)	
- Severe/immobile	58 (20.3)	48 (17.5)	
Cognitive impairment, *n* (%)			.193^b^
- Not restless or confused	127 (44.4)	136 (49.6)	
- Moderate restless or confused	136 (47.6)	110 (40.1)	
- *Very restless or confused*	23 (8.0)	28 (10.2)	
Number patients assessed for delirium, *n* (%)	73 (25.5)	55 (20.1)	.170^b^
- No delirium (DOSS ≤ 2)	57 (78.1)	37 (73.4)	
- Delirium (DOSS ≥3)	17 (21.9)	18 (26.6)	

*Note.* DOSS = Delirium Observation Screening Scale.

^a^ mean (*SD*). ^b^ χ^2^.

## Discussion

This part of the Erasmus MC WELCOME study reports the incidence of falls both before and after the move to a new hospital. Concerns that the fall rate would increase in the new hospital due to less social control, this was not confirmed. Furthermore, the incidence of delirium in patients who fell did not differ between the former and new hospital. It must be noted, however, that only 23% of the falls had been assessed for delirium. We expect that only patients with an increased risk of delirium were assessed. Thus, we could not ascertain whether staying in a single-occupancy room affects the risk of delirium.

The reported overall fall rate in our study was 1.4 per 1,000 patient days. This rate is relatively low compared to the reported fall rates in the literature, which range from 1.3 to 16.9 per 1,000 patient days depending on hospital setting or ward type (de Souza et al., 2019; Kisacik & Cigerci, 2019; Najafpour et al., 2019; Röhsig et al., 2019; Thomas & Balmforth, 2019). However, the phenomenon of underreporting could have contributed to the low fall rate. Underreporting is a well-known limitation of incident reporting systems. It is assumed that one in five incidents is reported (Mulac et al., 2020). On the other hand, screening for fall risk in frail patients has become common practice in Dutch hospitals after the implementation of National Safety Management System guidelines (Dutch Patient Safety Programme, 2013; van Schoten et al., 2018). Since 2018, a fall prevention committee in our hospital—including nursing, medical, and allied health staff—reviews the pre-existing fall prevention policies and procedures, monitors data, enhances staff awareness, and stimulates fall screening.

In hospital settings, the fall risk increases with age (Healey et al., 2008). In our study, 64% of the patients who fell were younger than 70 years. This finding suggests that in addition to the intrinsic factor older age, other intrinsic factors, such as chronic conditions (e.g., diabetes, COPD), medication consumption (e.g., antidepressants, benzodiazepines, and cerebral vasodilator), surgery, or mental status (e.g., cognitive impairment) are associated with in-hospital falls (Poh & Shorey, 2020). Therefore, we suppose falls may be caused by intrinsic patient factors rather than solely being the consequence of staying in a single-occupancy room.

Another finding of interest was the high proportion of falls in the before period, which were associated with getting up from bed, wheelchair, or chair. In the new hospital, all patient rooms are equipped with motorized ceiling hoists (opposed to one or no patient lifts in the wards of the former hospital). We assume that this could have had a positive effect on the incidence of falls during transfer from bed to chair. In the after period, the incidence of falls in bathrooms had nearly doubled (from 9.4% to 17.2%). In the former hospital, vulnerable patients pull the alarm cord if they need assistance. We suppose that patients in the new hospital, who were in the en-suite bathroom (e.g., toileting) and need assistance, did not always wear the wrist alarm. The literature shows that extrinsic factors related to the environment (e.g., lack of wall railings, obstructed walkways) represent major risk factors for falls (de Souza et al., 2019). In line with other studies, the majority of the falls were reported by nurses and were unwitnessed (Hignett et al., 2013; Hill et al., 2010).

## Delirium

The relationship between falls and delirium is complex. Falls may contribute to the development of delirium, and delirium may lead to a fall (Sillner et al., 2019). Delirium in patients who fell is often insufficiently screened, particularly in persons with preexisting dementia (Babine et al., 2018; Sillner et al., 2019). In our study, almost half of the patients who fell were reported as very restless and confused, although only one quarter of them had been screened for delirium (Duppils & Wikblad, 2004). Therefore, hospital staff should pay attention to this kind of behavioral changes and be aware that restlessness and confusion could affect patients’ safety. Nevertheless, delirium is a condition that fluctuates and thus may easily be missed by hospital staff (Sillner et al., 2019). We would recommend developing and validating a patient safety risk algorithm embedded in the electronic medical record and linked to incident reporting, including falls and delirium.

## Strengths and Limitations

A major strength of our study is that we included all types of hospital wards and populations, in contrast to most studies that have focused on specific hospital wards populations.

Several limitations of the study need to be considered. First, this was a single-center, retrospective study with a pragmatic focus to analyze only outcome data. Therefore, our findings must be interpreted with some caution because this is a comparative study without adjustment for confounding factors. Second, the study was conducted in a university hospital, which may impair generalizability of the findings to other types of hospitals with less complex patients. Third, given the retrospective design of this study, it was not possible to explore the changes to caregiver duties in the new hospital. Finally, underreporting is a well-known limitation of the incident reporting systems (Mulac et al., 2020). It is assumed that during the 2 months after the move slightly, fewer incidents were reported compared to the same period before the move because we found a considerably lower number of reported falls right after the move.

## Conclusion

Although concerns had been raised that the falls rate would increase due to less social control in single-occupancy patient rooms, this was not confirmed. However, it seems that more falls occur in the bathroom. The effects on patient safety related to design of healthcare environments needs further research.

## Implications for Practice

- Single-occupancy patient rooms—with less social control compared to multibedded rooms—do not seem to lead to an increased risk of falls. Efforts should be made to improve adherence to incident reporting as currently the real incidence of falls seems to be underreported.
- Because we found a high incidence of delirium among the fallers, we strongly advocate adequate delirium and fall risk assessment.
- Multidisciplinary teams need to collaborate when planning for building a new clinic with 100% single-occupancy patient rooms. By incorporating the competences of architects, designers, nurses, physicians, and former patients, those with expertise can work together, to provide the best possible result on patient safety issues (e.g., falls and sleep quality) and staff and patients’ perceptions.

## Supplemental Material

Supplemental Material, sj-pdf-1-her-10.1177_19375867221123607 - Falls Incidence Compared Between a Multibedded Ward Hospital and a 100% Single-Occupancy Room Hospital: An Uncontrolled Before-After Study Click here for additional data file. Supplemental Material, sj-pdf-1-her-10.1177_19375867221123607 for Falls Incidence Compared Between a Multibedded Ward Hospital and a 100% Single-Occupancy Room Hospital: An Uncontrolled Before-After Study by Fozia Hussain, Monique van Dijk, Christian Oudshoorn and Erwin Ista in HERD: Health Environments Research & Design Journal
