# Supplementary material for: Falls Incidence Compared Between a Multibedded Ward Hospital and a 100% Single-Occupancy Room Hospital: An Uncontrolled Before-After Study
Source: HERD. 2022 Sep 7;16(1):131–41. doi: 10.1177/19375867221123607 (PMC9755692; doi:10.1177/19375867221123607)
Supplement: Supplemental Material, sj-pdf-1-her-10.1177_19375867221123607 - Falls Incidence Compared Between a Multibedded Ward Hospital and a 100% Single-Occupancy Room Hospital: An Uncontrolled Before-After Study [file sj-pdf-1-her-10.1177_19375867221123607.pdf]

Appendix:

Appendix 1: Photos of patients’ rooms and nursing station (former and new building)

Appendix 2: Description of the categories of severity of fall-related injury

Appendix 3: Delirium Observation Screening Scale

Appendix 1 Photos patient rooms:

Four-beds patient room (old hospital)

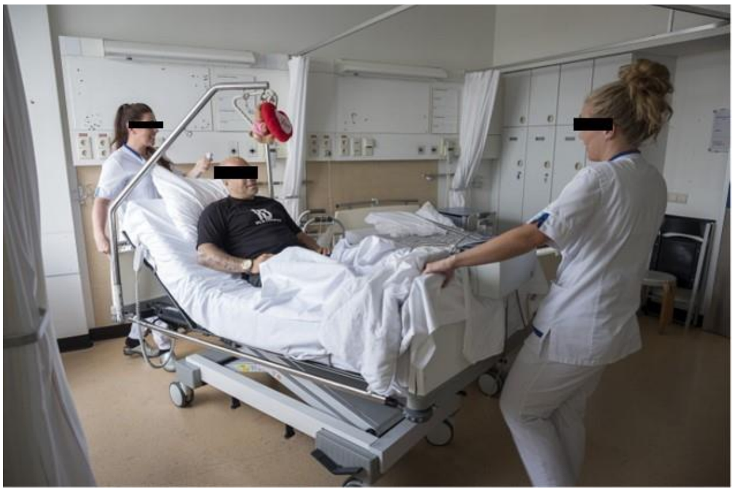

Single patient room (new hospital)

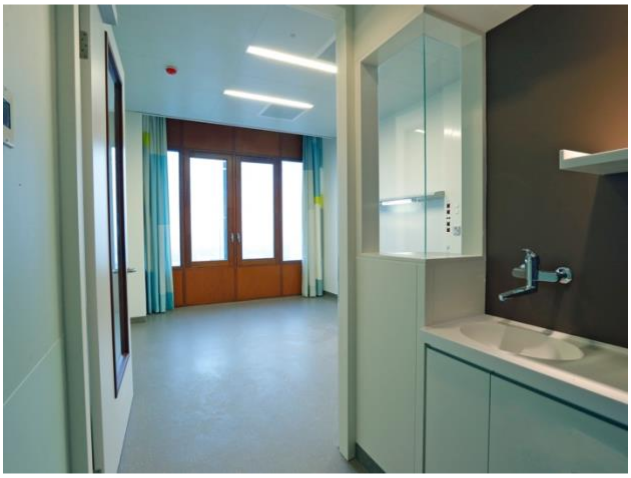

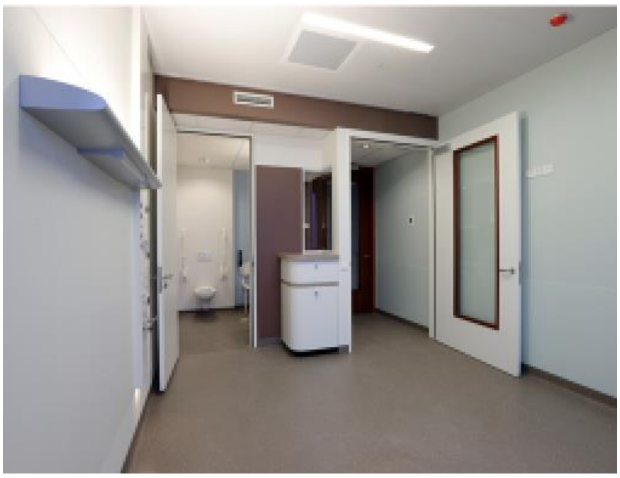

Decentralized Nursing station (new hosptial)

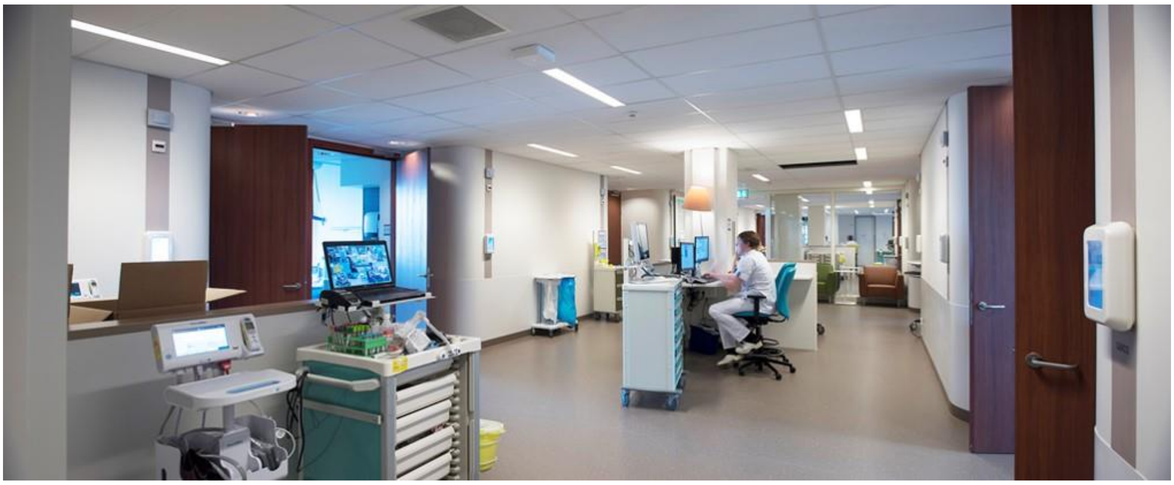

Appendix 2: Description of the categories of severity of fall-related injury

Category	Description
No harm	No visible injuries or complaints
Low (minimal)	Hematoma or wound but no specific complaints
Moderate	Wound and/or contusion with pain and/or short-term functional limitation due to the wound, contusion or pain (and sometimes medication in the form of pain relief)
Severe	Fracture/intracranial injury where surgery or intensive care admission is indicated. Or serious functional consequences
Death	Death caused by fall

Appendix 3: Delirium Observation Screening Scale

The working method of the DOS scale.
The patient
1. Dozes off during conversation or activities<br>2. Is easily distracted by stimuli from the environment<br>3. Maintains attention to conversation or action<br>4. Does not finish question or answer<br>5. Gives answers that do not fit the question<br>6. Reacts slowly to instructions<br>7. Thinks to be somewhere else<br>8. Knows which part of the day it is<br>9. Remembers recent events<br>10. Is picking, disorderly, restless<br>11. Pulls IV tubes, feeding tubes, catheters etc.<br>12. Is easily or suddenly emotional (frightened, angry, irritated)<br>13. Sees/hears things which are not there
Never=0 points; Sometimes or always=1 point.
A total score of three or more points indicate a delirium.
